# Atypical presentation of an obstructed obturator hernia in a 99-year-old female: a case report

**DOI:** 10.1093/jscr/rjac174

**Published:** 2022-04-27

**Authors:** Ngozi Anyaugo, Obisike Nwogwugwu, Chiara Rossi, Zara Toney

**Affiliations:** Department of General Surgery, Weston General Hospital, Grange Road Uphill, Weston-super-Mare, University Hospitals Bristol and Weston NHS Trust, UK; Department of General Surgery, Weston General Hospital, Grange Road Uphill, Weston-super-Mare, University Hospitals Bristol and Weston NHS Trust, UK; Department of General Surgery, Weston General Hospital, Grange Road Uphill, Weston-super-Mare, University Hospitals Bristol and Weston NHS Trust, UK; Department of General Surgery, Weston General Hospital, Grange Road Uphill, Weston-super-Mare, University Hospitals Bristol and Weston NHS Trust, UK

## Abstract

An obturator hernia is the protrusion of an organ/tissue through the obturator canal. Fondly called ‘little old lady’s hernia’, as they typically affect elderly thin female patients [7]. They are rare and difficult to clinically diagnose [2]. Diagnosis is often delayed and presentation could vary from symptoms of bowel obstruction, and pain in the groin or medial thigh [6] to atypical presentations like in our case. We report a case of a 99-year-old female with a 3-day history of low back pain, nausea and constipation. Computerized tomography scan revealed small bowel obstruction with transition point in left obturator hernia. The obstruction was successfully relieved via surgery without the need for bowel resection. This case highlights the importance of a high index of suspicion when faced with vague symptoms. Obturator hernias carry a reasonable degree of morbidity and mortality without intervention but have good outcomes if promptly managed.

## INTRODUCTION

Obturator hernias are rare and difficult to clinically diagnose [2]. Medical literature reports the incidence of obturator hernias to be between 0.05 and 2.2% of all hernias [2].

The mortality rate of obturator hernia is the highest among all abdominal wall hernias (13–40%) if untreated [11]. Higher mortality rates of 47.6 have been described in some literature [5]. Early diagnosis and surgical treatment contribute significantly to reduce the mortality and morbidity rates.

## CASE REPORT

A 99-year-old lady presented at the Accident and Emergency department with a 3-day history of worsening lower back pain, decreased mobility, nausea, constipation and some urinary incontinence. She additionally reported right-sided flank pain, appeared dehydrated but with normal observations.

An initial diagnosis of urinary tract infection was made and appropriate antibiotics prescribed as per the hospital guidelines. Medical history included hypertension, haemorrhoids, COVID vaccinations and presbycusis. Her routine medications were antihypertensive and cholecalciferol. Physical examination on admission revealed a soft, distended abdomen and an abdominal X-ray which showed dilated loops of small bowel was done due to the medical team’s worry about her physical examination findings.

**Figure 1:**
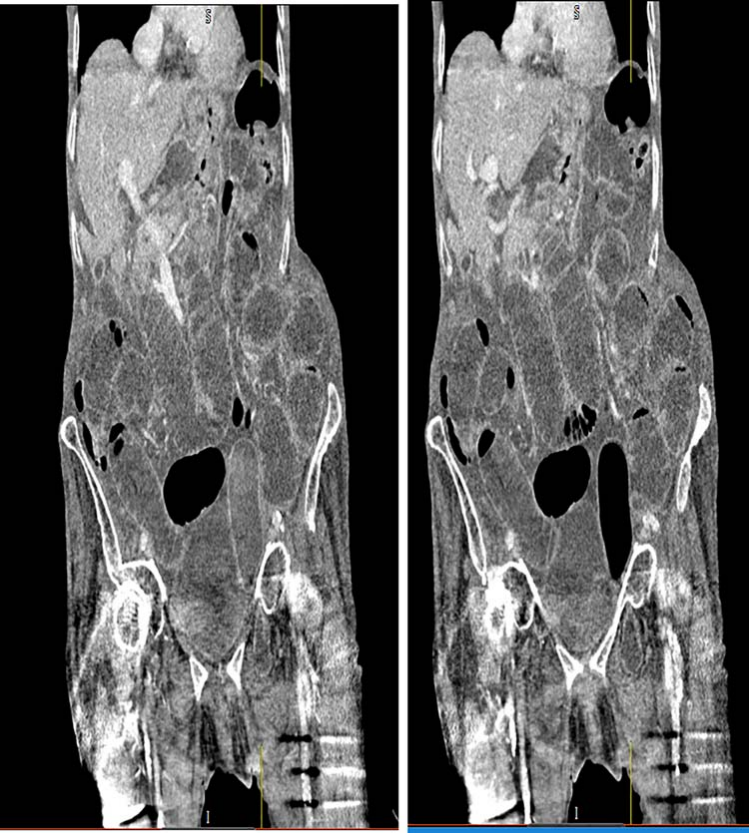
Coronal view CT scan with IV contrast showing dilated small bowel loops and bowel loop through the left obturator canal.

**Figure 2:**
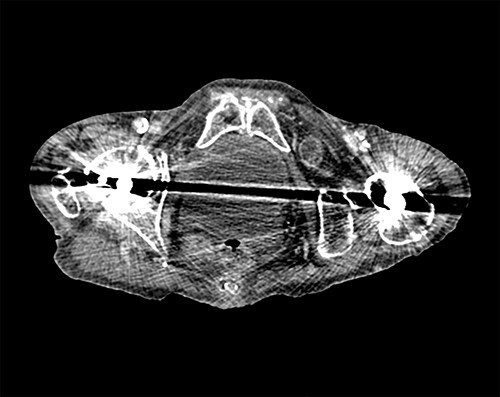
Axial view of CT scan showing the bowel loop through the left obturator hernia.

Following surgical review, a computerized tomography (CT) scan of the abdomen and pelvis was requested and showed small bowel obstruction, secondary to a left-sided obturator hernia (Figs 1 and 2).

Explaining the diagnosis, intended procedure and obtaining informed consent were challenging due to the patient’s significant hearing loss. The risks of the procedure were, however, discussed, and the patient agreed to surgery. Her estimated mortality using the National Emergency Laparotomy Audit risk calculation tool was 8.2% and Portsmouth Physiological and Operative Severity Score for the Enumeration of Mortality risk for morbidity was 62.9%. A multidisciplinary team meeting between the medical, surgical and anaesthetic teams was held to discuss ceilings of care and a consensus to go ahead was reached considering her frailty and age.

A lower midline laparotomy incision was done and opened to the peritoneum. Operative findings included some serous fluid in peritoneum, an incarcerated ileal loop in a left obturator hernia and distension of small bowel proximal to this point. The affected bowel loop was bruised and congested, but not ischaemic. Careful release of the obstructed hernia followed, with the released bowel loop warmed for 5 min. After warm saline lavage, Surgicel (oxidized regenerated cellulose) was placed into the pelvis to plug the hernia space. Mass closure was performed with polydioxanone suture (PDS) and the skin was closed with staples.

The patient had an uneventful post op recovery and suffered no complications. She was surgically fit for discharge by Day 8 post-operatively but needed transfer to a rehabilitation facility to aid recovery to her pre-admission level of physical functioning.

## DISCUSSION

Clinical signs may be non-specific in the absence of signs relating to obturator nerve compression [1]. The Howship Romberg sign, which is when pain is elicited along the distribution of the obturator nerve due to compression of the nerve, is only present in 15–50% of patients [9]. Another sign described is the Hannington-Kiff sign in which there is an absent adductor reflex in the thigh in the presence of a positive patellar reflex. More importantly, a high index of suspicion is needed in elderly females presenting with clinical signs of small bowel obstruction.

In our case, her presenting symptoms of low back pain and decreased mobility provided no direction towards the right diagnosis. However, her abdominal examination and abdominal X-ray findings were suggestive of small bowel obstruction [8].

The accuracy of CT scan with IV contrast in diagnosing obturator hernia is $\sim$90% [3, 4]. This makes it the imaging modality of choice and even so, there are cases where the obturator hernia can be missed on CT [10].

In elderly frail patients, there is always an ongoing dilemma of balancing risk of surgical intervention versus benefits. Our case presented a similar dilemma which was promptly resolved by a best interest meeting.

Open and laparoscopic approaches can be used, but in the emergency setting, an open laparotomy might be a more sensible option. This is due to technical difficulty of performing laparoscopic surgery on an obstructed bowel due to limited intraabdominal space [12].

There are several open operative approaches described for the repair of obturator hernia. These include the abdominal, retropubic, obturator and inguinal approaches. In the emergency setting, the abdominal approach via a low midline incision is most commonly favoured, as it allows adequate exposure of the obturator ring as well as the identification and resection of any ischaemic bowel [12].

The open approach was suitable in this circumstance, bowel resection was not indicated as the intervention was timely and bowel ischaemia had not occurred.

Following reduction of the hernia, the hernia can be repaired using several techniques. There are several repair techniques that have been described: sac ligation alone, direct suture repair, use of autologous tissue or prosthetic repair. Many authors prefer a simple closure of the hernia defect if visible with one or more interrupted sutures as it leads to an acceptable recurrence rate of <10% [11]. In our case, the plug technique [13] using a Surgicel plug over the obturator canal was utilized.

Despite many papers dealing with various surgical techniques used in the treatment of obturator herniae, it is important to emphasize diagnosis and early intervention is the most important aspect of management [14].

Possible sequela of the untreated obstructed obturator hernia includes bowel ischaemia, perforation and faecal peritonitis. This significantly increases mortality.

Whatever the approach, the emphasis should be on rapid evaluation, adequate resuscitation and early operative intervention to reduce morbidity and mortality [11].

For the purposes of achieving a prompt accurate diagnosis, a CT scan is indispensable. Majority of patients with symptomatic obturator hernia are old and frail, with multiple comorbidities. Without operative intervention, these patients will likely die. However, with appropriate discussion, a 99-year-old can have a successful post op recovery from a laparotomy and open repair of an obstructed obturator hernia.

Informed consent was obtained and all patient identifiable data anonymized in this report.
